# Direct synthesis of nanostructured silver antimony sulfide powders from metal xanthate precursors

**DOI:** 10.1038/s41598-021-82446-3

**Published:** 2021-02-04

**Authors:** Yasser T. Alharbi, Firoz Alam, Abdelmajid Salhi, Mohamed Missous, David J. Lewis

**Affiliations:** 1 Department of Chemistry, The University of Manchester, Oxford Road, Manchester, M13 9PL UK; 2 Department of Materials, The University of Manchester, Oxford Road, Manchester, M13 9PL UK; 3 Department of Electrical and Electronic Engineering, The University of Manchester, Sackville Street, Manchester, M13 9PL UK

**Keywords:** Chemistry, Materials science

## Abstract

Silver(I) ethylxanthate [AgS_2_COEt] (**1**) and antimony(III) ethylxanthate [Sb(S_2_COEt)_3_] (**2**) have been synthesised, characterised and used as precursors for the preparation of AgSbS_2_ powders and thin films using a solvent-free melt method and spin coating technique, respectively. The as-synthesized AgSbS_2_ powders were characterized by powder X-ray diffraction (XRD), Raman spectroscopy, scanning electron microscopy (SEM) and energy dispersive X-ray (EDX) spectroscopy. The crystalline AgSbS_2_ powder was investigated using XRD, which shows that AgSbS_2_ has cuboargyrite as the dominant phase, which was also confirmed by Raman spectroscopy. SEM was also used to study the morphology of the resulting material which is potentially nanostructured. EDX spectra gives a clear indication of the presence of silver (Ag), antimony (Sb) and sulfur (S) in material, suggesting that decomposition is clean and produces high quality AgSbS_2_ crystalline powder, which is consistent with the XRD and Raman data. Electronic properties of AgSbS_2_ thin films deposited by spin coating show a p-type conductivity with measured carrier mobility of 81 cm^2^ V^−1^ s^−1^ and carrier concentration of 1.9 × 10^15^ cm^−3^. The findings of this study reveal a new bottom-up route to these compounds, which have potential application as absorber layers in solar cells.

## Introduction

Considerable research attention has been focused on the application of binary, ternary and quaternary chalcogenides as absorber layers in thin film solar cells^1–3^. Metal chalcogenides have gained interest due to their potential in ferroelectric, thermoelectric devices and for their non-linear optical properties^4–11^. Copper indium gallium selenide (CIGS) and cadmium telluride (CdTe) are the most commonly used light-absorbing materials in thin film solar cell^12^. However, low cost, earth-abundant and cadmium-free materials can potentially be used as an alternative^13^.

The creation of inorganic ternary materials containing three elements is desirable due to the range of possible new materials with novel electronic properties. As such, there has been significant scientific interest in the synthesis of such materials, and more specifically, ternary chalcogenides, for the fabrication of highly efficient, cheap and environmentally friendly photovoltaic devices^14^. Such ternary materials can be produced by mixing elements from different groups of the periodic table such as NiCo_2_S_4_^15^ and Ag_8_SnS_6_^16^. Focusing on I–III–VI_2_-type and I–III_2_–VI_4_-types, which include elements from group I (Cu, Ag), group III (Ga and In) and group VI (S and Se) results in chalcopyrite-type materials. These are desirable due to their reduced toxicity, and high absorption coefficients extending across the visible to near-infrared wavelengths ^17^.

A variety of compounds, including CuSbS_2_ (Eg = 1.5 eV), SnS (Eg = 1.1 eV), Cu_2_SnS_3_ (Eg = 1.15 eV) AgSbSe_2_ (Eg = 1 eV) and AgSbS_2_ (Eg = 1.7 eV) have desirable optical properties for solar cell applications, mainly due to their bandgap commensurate with AM 1.5G photon flux maxima^18–22^.

**Figure 1:**
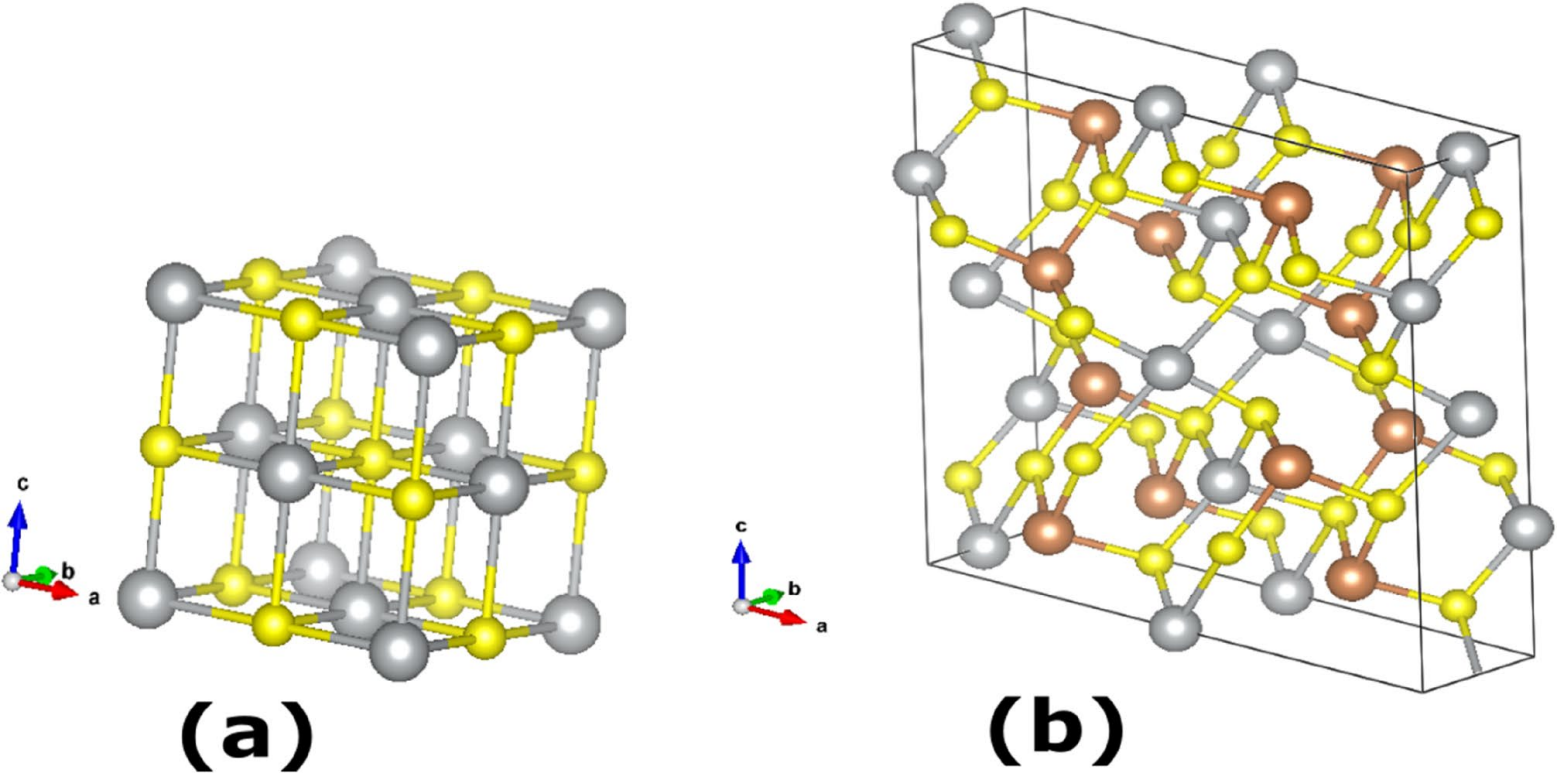
(**a**) Crystal structure of cubic cuboargyrite β-AgSbS_2_ as reported by Geller and Wernick, with the silver and antimony indistinguishable^33^. (**b**) Crystal structure of monoclinic α-AgSbS_2_ miargyrite as obtained by Smith^34^; silver atoms (Ag) are represented by silver spheres, Antimony (Sb) by brown spheres and S by yellow spheres.

Interest in chalcogenides such as AAsSe_2_ (A = Li, Na) and AgSbEQ_2_ (EQ = S, Se) has predominantly been due to the distinct ferroelectric, thermoelectric and non-linear optical properties they present^23–26^. Alloys consisting of AgSbSe_2_ have potential use in solar cells due to their high optical-absorption coefficient of > 10^4^ cm^−1^ across the Vis–NIR region of the electromagnetic spectrum and band gap energies of 0.9–1.1 eV, which can maximise the theoretical power conversion efficiency according the Shockley–Queisser limit (ca. 24% at these values of E_g_)^27–29^. Usually, AgSbSe_2_ crystallises in the halite structure, whereby Ag and Sb randomly occupy the crystallographic Wyckoff positions^30^. The AgSbS_2_ ternary chalcogenide may be modified to give rise to quaternary compounds of the form (MS)_1−x_ (AgSbS_2_)_x_ (M = Ge, Sn, Pb), which represent a family of semiconductors and semi-metals with low to narrow optical band gap energies in the range 0.01–0.6 eV^31,32^. At elevated temperatures, AgSbS_2_ exists as cubic β-AgSbS_2_^33^, whilst at low temperatures (˂380 °C) monoclinic α-AgSbS_2_^33^ is the dominant phase (Fig. 1) ^33–35^. The crystalline structure of α-AgSbS_2_ has been studied over a number of decades by a series of authors, including Hofmann (1938)^36^, Knowles (1964)^37^ and Smith et al., (1997)^34^. Effenberger et al. (2002) demonstrated that the structure is comprised of pyramids of SbS_3_ and chains linked by linear S–Ag–S and AgS_4_ polyhedra^38^.

A variety of techniques have been established for the formation of AgSbS_2_ thin films including thermal evaporation^39^ , pulsed-laser deposition^40^, RF-magnetron sputtering^41^ and laser ablation^42^. For each of the above techniques the starting material is prepared by direct fusion of stoichiometric quantities of the elements, which can be problematic due to the formation of sub phases caused by inequivalent ion migration rates in the solid state. The use of metal xanthate precursors, however, may circumvent this problem as the mixing prior to thermal decomposition occurs at the nanoscale and hence final products should be homogeneous and of a single crystalline phase, with the bottom up nature of the process allowing for exquisite control of elemental constitution. Due to the pre-formed bonds between metal and chalcogenide atoms, metal xanthates can act as efficient precursors for the formation of solid state metal sulfides. This has led to the extensive application of, for instance, xanthate complexes for the production of thin films^43,44^. Advantages conferred by this sort of synthetic route include the ability to carry out low temperature decomposition, the ease of synthesis and stability of the resulting compound in air, along with the fact that by-products for these materials are generally gaseous. O’Brien & Lewis have reported a number of such syntheses for a range of main group and transition metal sulfides^45–50^.

In this paper, we describe a metal xanthate precursor route to produce ternary silver antimony sulfide (AgSbS_2_) as a single well-defined phase via thermal decomposition of metal xanthate precursors in stoichiometric ratios. AgSbS_2_ is rarely found in nature but possesses potentially excellent properties for solar cell applications^51,52^.

## Results and discussion

Metal xanthate complexes of the form [AgS_2_COEt] (**1**) and [Sb(S_2_COEt)_3_] (**2**) were synthesised via metathesis reactions of the nitrate/chloride metal salts with potassium ethyl xanthate^52–54^. Infrared (IR) and nuclear magnetic resonsnce (NMR) spectroscopies were used to assess the purity of complexes (**1**) and (**2**) and the spectra recorded are shown in the ESI (Fig. S1.1 and S1.2). The Ag_2_S and Sb_2_S_3_ powders synthesised from [AgS_2_COEt] **(1)** and [Sb(S_2_COEt)_3_] **(2)** at three different temperatures (400 °C, 450 °C and 500 °C) were then characterised using XRD shows a pure phase Acanthite Ag_2_S (Fig. S2.1) and stable Stibnite phase of Sb_2_S_3_ (Fig. S2.2), respectively. The Raman spectra of both metal sulfide (Ag_2_S and Sb_2_S_3_) synthesised from precursors (**1**) and (**2**) at three different temperatures (400 °C, 450 °C and 500 °C) are shown in Figs. S2.3 and S2.4, respectively.

### Thermogravimetric analysis of [AgS_2_COEt] (1) and [Sb(S_2_COEt)_3_] (2) complexes

**Figure 2:**
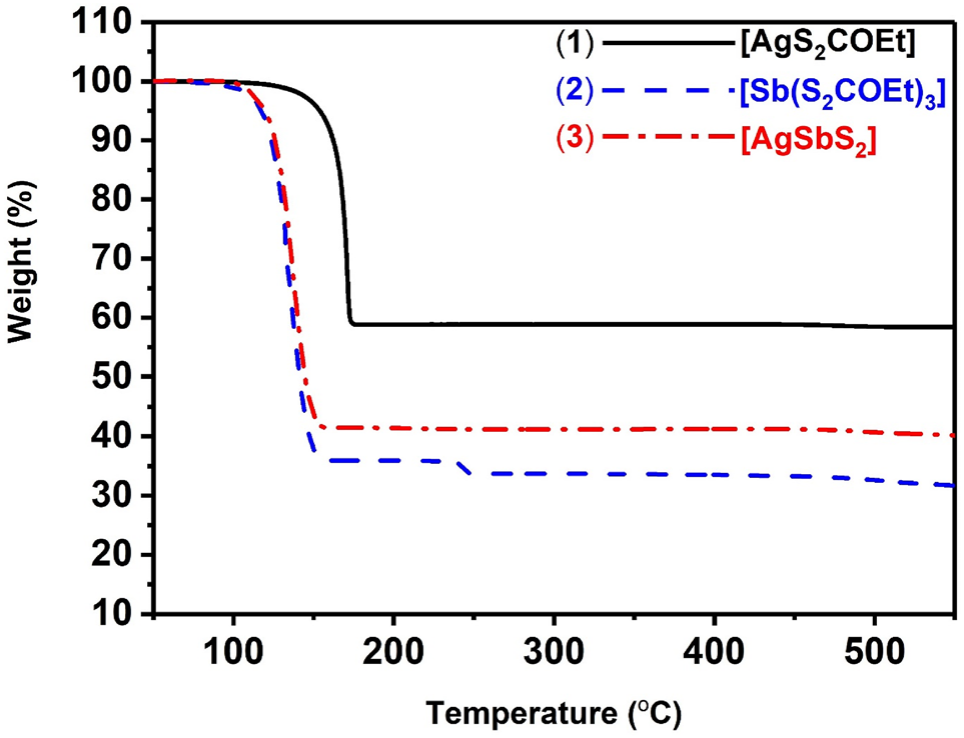
Thermogravimetric analysis (TGA) profiles of (**1**) [AgS_2_COEt], (**2**) [Sb(S_2_COEt)_3_], and (**3**) mixtures of Ag and Sb xanthates to form AgSbS_2_.

Thermogravimetric analysis of (**1**) and (**2**) was performed in the temperature range of 0 °C to 550 °C under a nitrogen atmosphere. Both complexes exhibited a large mass loss between 80 and 250 °C (Fig. 2). The decomposition of **(1)** started at 96 °C and ended at 177 °C with the remaining weight determined to be 54%, which is matching the calculated value of 54%. Both experimental and theoretical values confirmed the phase of Ag_2_S. In a similar manner, the TGA profile of **(2)** exhibits the main decomposition step between 100 and 161 °C. The final residue of 35% is in good agreement with the calculated value of 35% which confirms the formation of Sb_2_S_3_. The minor decomposition step with < 3% mass loss is attributed to loss of sulfur which was also observed by Alqhatani *et al*^54^. TGA of mixtures of the two complexes (Fig. 2) shows a single step decomposition at ca. 200 °C with a remaining weight of 41% which corresponds to the formation of AgSbS_2_. This low temperature decomposition of the complexes to produce AgSbS_2_ means that it could potentially be produced within polymer matrices and can be used as an absorber layers in polymer nanocrystal based hybrid solar cells^55–57^. O’Brien et al. has reported the preparation of PbS nanocrystals in polymer matrix via decomposition of lead(II) xanthates in polystyrene matrices as a potential absorber material for flexible hybrid photovoltaic devices^58^.

**Scheme 1:**
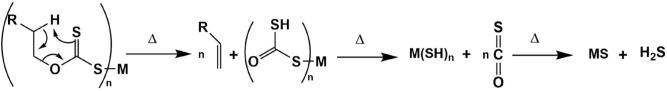
Metal xanthate pyrolysis by the Chugaev elimination mechanism to produce metal sulfide MS^62,63^.

The mixtures of the solid precursors form a homogenous molten intermediate reactive melt, when the temperature increased. Before undergoing decomposition to form the final solid products. The volatile organic components are evacuated through the constant nitrogen flow^53,59,60^. The mechanism of xanthate decomposition follows a Chugaev elimination reaction which involves the production of a cyclic transition state to produce carbonyl sulfide molecules (OCS) and alkenic side products (Scheme 1)^61,62^. Alanazi et al. has previously reported the synthesis of stannite Cu_2_FeSnS_4_ (CFTS) quaternary chalcogenides from mixtures of metal (O-ethylxanthato) (M = Cu, Fe and Sn) complexes, which shows that combining xanthate precursors in tandem in reactive melts is a promising approach to these materials^63^.

**Figure 3:**
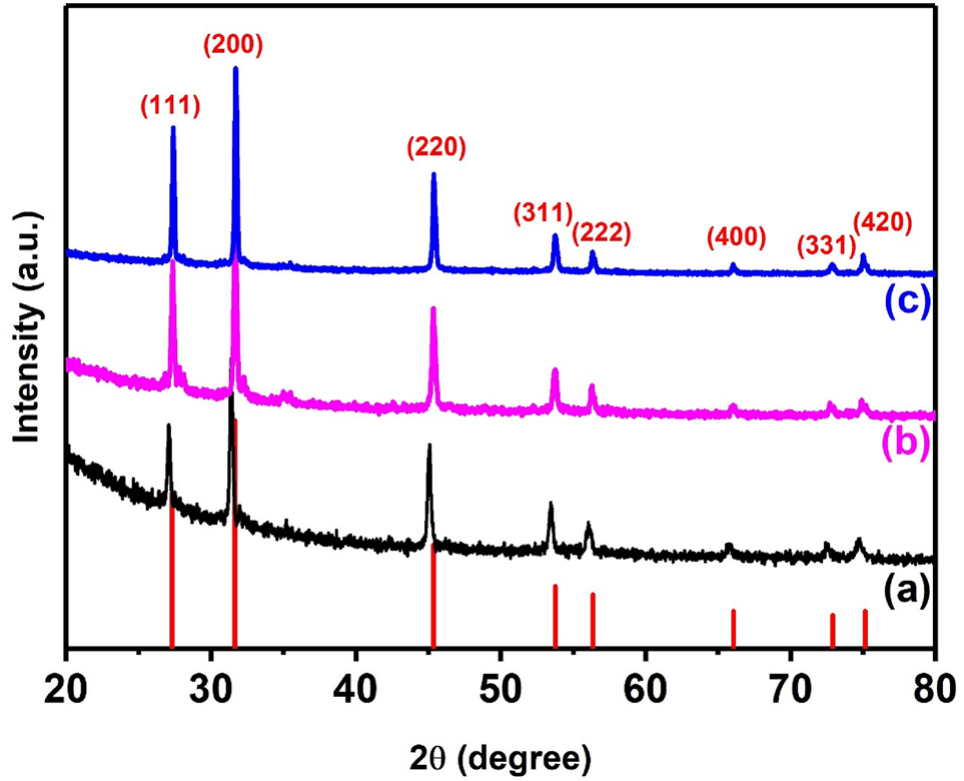
XRD patterns of AgSbS_2_ powders synthesised at various temperatures (**a**) 400 °C (**b**) 450 °C, and (**c**) 500 °C for 1 h under nitrogen. The red sticks correspond to the standard powder diffraction pattern of cubic AgSbS_2_ (cuboargyrite, ICDD No. 00-017-0456).

**Figure 4:**
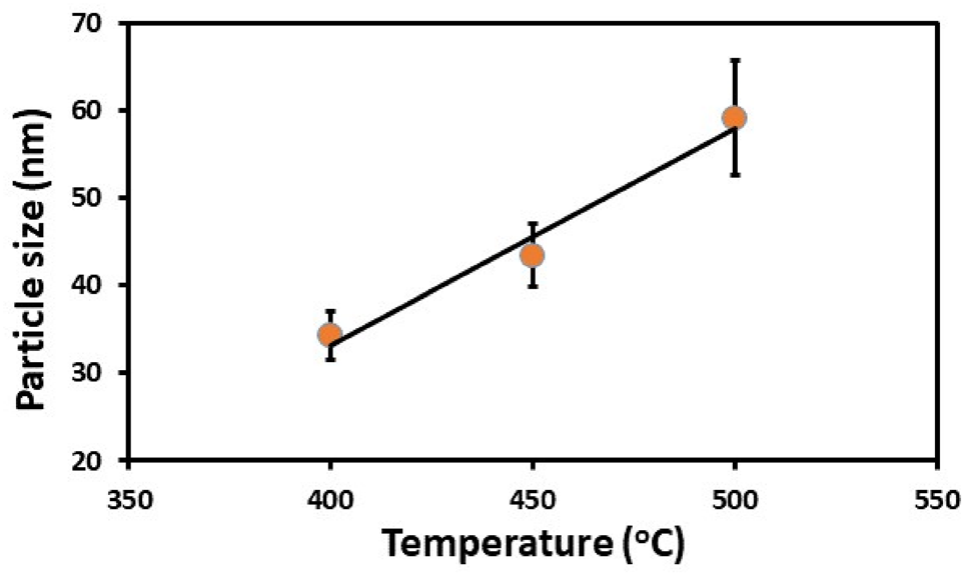
Average crystallite domain size of AgSbS_2_ powders calculated using Scherrer’s equation as a function of synthesis temperature.

Therefore, pyrolysis of mixtures of Ag and Sb precursors was carried out at various temperature such as (300 °C, 350 °C 400 °C, 450 °C and 500 °C). The powder XRD pattern of AgSbS_2_ powders synthesised at 300 and 350 °C have some impurity peaks as shown in ESI (Fig. S2.5). The XRD powder pattern of polycrystalline AgSbS_2_ powders synthesised at 400 °C can be ascribed to cubic AgSbS_2_ (cuboargyrite, ICDD No. 00-017-0456, space group [Fm-3 m] and *a* = 5.6520 Å) with Bragg peaks at 2θ = 27.4°, 31.7°, 45.3°, 53.7°, 56.7°, 66.0°, 72.8°, 75.0° that could be indexed to the (1 1 1), (2 0 0), (2 2 0), (3 1 1) (2 2 2) (4 0 0) (3 3 1) and (4 2 0) planes respectively (Fig. 3). The positions of the peaks in Fig. 3a (black line) are shifted toward smaller 2θ values with respect to those observed in Fig. 3b and c. Since all the peaks are shifted by same 2θ value, it is likely that this is a measurement error associated with the height of the sample in the diffractometer. Additionally, we have also observed that the peaks become more intense and the FWHM of each peak is reduced when the synthesis temperature was increased from 400 to 500 °C. The average crystallite domain size of AgSbS_2_ powders synthesised at 400 °C, 450 °C and 500 °C are 34 nm, 43 nm and 59 nm respectively, as calculated using Scherrer’s equation^64^ (Fig. 4). The crystallite domain size found in AgSbS_2_ powders increases with increasing synthesis temperature, which is in agreement with previously reported data^65–67^.

**Figure 5:**
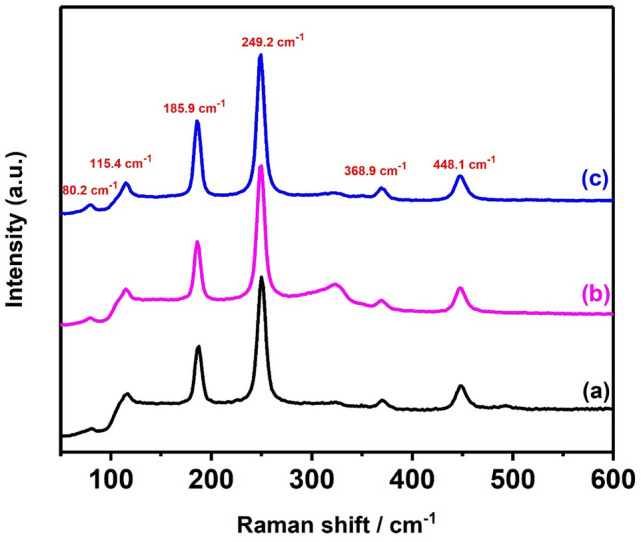
Raman spectra of AgSbS_2_ powders synthesised at different temperatures (**a**) 400 °C, (**b**) 450 °C and (**c**) 500 °C for 1 h under nitrogen.

Raman spectroscopy was conducted on the AgSbS_2_ powder produced at 500 °C (Fig. 5). Raman resonances are observed at 80.2, 115.4, 185.9, 249.2, 368.9 and 448.1 cm^−1^ respectively, and the spectral positions of these peaks agree with those reported previously for AgSbS_2_^68^. The electrical properties of AgSbS_2_ films were measured using the Van Der Pauw method. Silver paste was used to form the four contacts on 7 × 7mm^2^ sample area. The measured carrier mobility and carrier concentration are 81 cm^2^ V^−1^ s^−1^ and 1.9 × 10^15^ cm^−3^, respectively. These values are comparable to values obtained for Cu_2_FeSnS_4_ (CFTS) films^63^. Hall effect measurements revealed that the films exhibit p-type conductivity.

**Figure 6:**
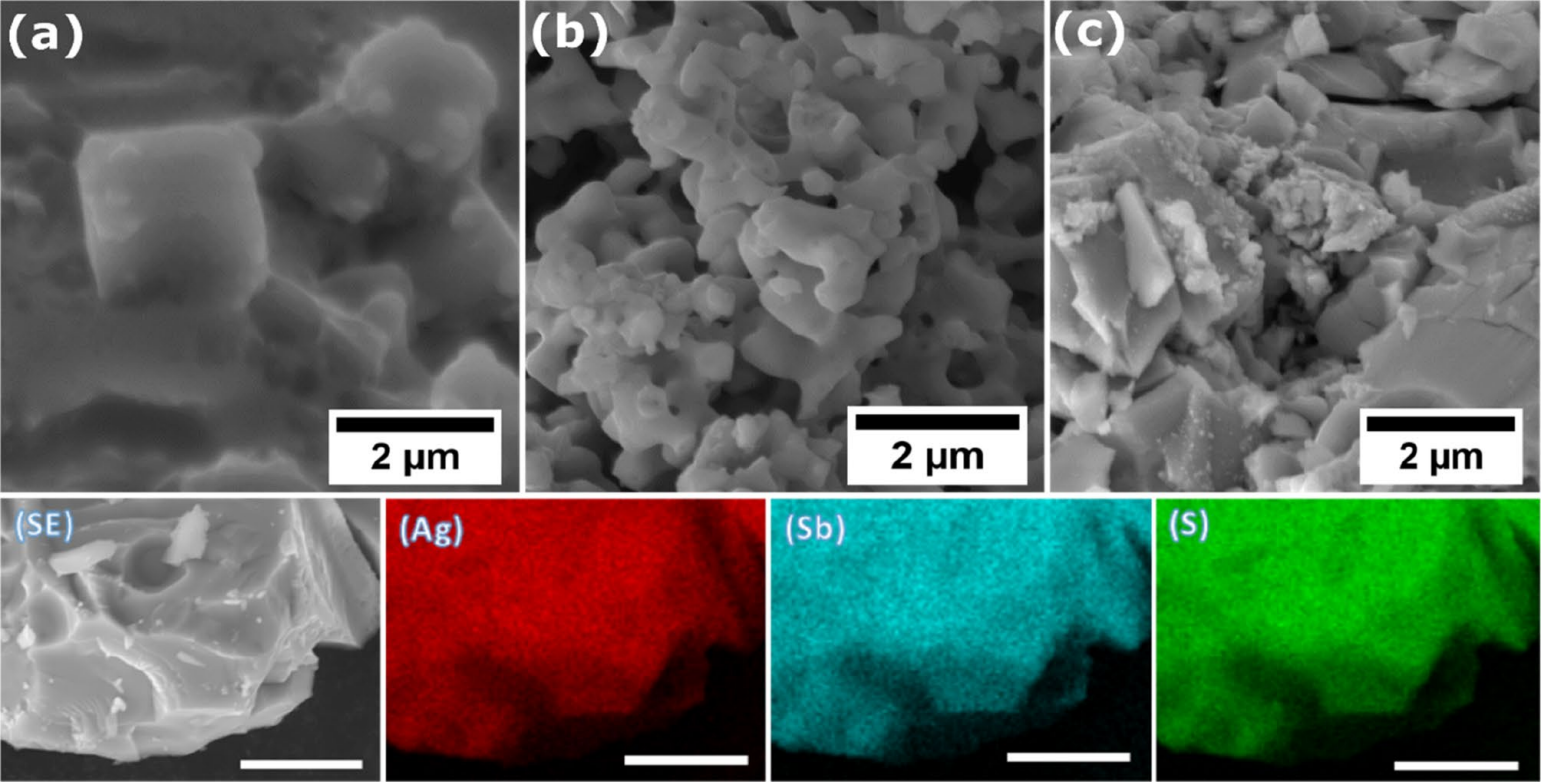
Top: SEM images of AgSbS_2_ powders produced at (**a**) 400, (**b**) 450 and (**c**) 500 °C, respectively. Bottom: EDX elemental maps revealing the distribution of Ag, Sb and S elements at the microscale for AgSbS_2_ produced at 500 °C (Ag Kα, Sb Kα and S Kα). The unlabelled scale bars represent 10 µm.

Secondary electron scanning electron microscopy (SEM) was used to interrogate the surface morphologies of the powders produced at different temperatures. Cubic structures are revealed for powders produced at 400 °C which changed to a porous appearance when the temperature of the synthesis was increased to 450 °C. When the temperature was increased to 500 °C, the morphology changed to flakes as shows in Fig. 6 and ESI (Fig. S2.6). Influence of the increasing temperature on the crystal structure has been reported by Habe et al.^69^. The AgSbS_2_ powders prepared at 400, 450 and 500 °C were analysed using energy-dispersive X-ray (EDX). EDX mapping gives information on the spatial distribution of elements at the micro to nanoscale and to ensure that the distribution of elements is homogeneous. Representative elemental mapping (Fig. 6) of these components showed a uniform distribution of the silver, antimony and sulfur. EDX spectra show that the samples consist only of the elements silver, antimony and sulfur, suggesting that decomposition is clean and produces high quality crystalline materials which is consistent with the XRD and Raman data from the same materials (see supporting information for EDX sum spectra Figs. S2.7 to S2.11).

## Conclusions

A novel, efficient and low temperature method for the synthesis of AgSbS_2_ powders has been demonstrated. Silver(I) ethylxanthate [AgS_2_COEt] **(1)** and antimony(III) ethylxanthate [Sb(S_2_COEt)_3_] **(2)** precursors have been used to produce crystalline powders of AgSbS_2_ with a high degree of atom efficiency. Ternary cubic AgSbS_2_ (cuboargyrite) was successfully produced which was evidenced by XRD and Raman spectroscopy. XRD data shows that crystallite size increase with increasing synthesis temperature. SEM images show a change in the surface morphology of these powders from cubic crystallites to flakes upon increasing the synthesis temperature. EDX mapping gives a clear indication of the presence of spatially co-localised Ag, Sb and S with no other elemental impurities. Use of solvents can be avoided altogether through the melt method which has great potential for the mass production of nanocrystalline powders of ternary materials.

## Methods

Methanol (99.8%), silver nitrate (99.9%), antimony (III) chloride (99%), potassium ethyl xanthate (96%) and chloroform-d (99%). All chemicals were purchased from Sigma-Aldrich and used as received.

### Synthesis of [AgS_2_COEt] (1)

Silver nitrate (1.7 g, 10.0 mmol) was dissolved in 20.0 ml of deionised water. The solution was added dropwise to aqueous potassium ethyl xanthate (2.0 g, 10.6 mmol) with a constant stirring for 40 min at room temperature. The silver ethyl xanthate precursor rapidly forms. A shiny green solid of the title compound was obtained by filtration and dried at room temperature. Yield 1.9 g (86%). Melting point (M.p). 150–154 °C Anal. calc. for AgS_2_COC_3_H_5_ (%): C 15.73, H 2.20, S 27.94, Ag 47.14. Found: C 15.84, H 2.13, S 27.89, Ag 46.91. FT-IR solid (ν_max_/cm^−1^): 2978.03 (w), 2938.5 (w), 1472.38 (w), 1355.23 (m), 1184.08 (s), 1136.83 (s), 1012.45 (s), 995.57 (s). ^13^C NMR: σ 227.55 ppm (S_2_C), σ 69.49 ppm (CH_2_) and σ 13.92 ppm (CH_3_).

### Synthesis of [Sb(S_2_COEt)_3_] (2)

Precursor (**2**) was prepared as per complex (**1**), but with antimony trichloride (2.0 g, 8.7 mmol) dissolved in 20 ml of methanol. The resulting solution was added dropwise to potassium ethyl xanthate (4.2 g, 26.2 mmol) which was dissolved in 80 ml of methanol. The crude product was isolated by filtration and recrystallized from chloroform to give pale yellow crystals. Yield: 3.5 g (80%). M.p. 88–92 °C. Anal. Calc. for Sb(S_2_COEt)_3_ (%) : C 22.28, H 3.12, S 39.58, Sb 25.12. Found: C 22.11, H 2.87, S 39.54, Sb 24.46. FT-IR solid (ν_max_/cm^−1^): 2988.64 (w), 2938.50 (w), 1468.05 (w), 1359.05 (w), 1186.49 (w), 1109.83 (s), 993.64 (s). ^13^C NMR: σ 222.49 ppm (S_2_C), σ 72.07 ppm (CH_2_) and σ 13.90 ppm (CH_3_).

### Synthesis of AgSbS_2_ powders

A homogenised mixture of [AgS_2_COEt] (**1**) and [Sb(S_2_COEt)_3_] (**2**) complexes (1:1 mol ratio) was placed in a ceramic boat that was subsequently placed in the centre of a glass tube which was then inserted into a Carbolite tube furnace. One end of the glass tube was directly connected to nitrogen gas through a Schlenk line in the fume hood, and the other end of the tube was carefully sealed with a rubber septum. A vacuum was used to remove any oxygen from the glass tube, and the glass tube was then refilled with nitrogen gas. After that the mixture was heated in the Carbolite furnace at 400 °C, 450 °C and 500 °C, respectively and kept it at each temperature for 1 h under nitrogen atmosphere to produce AgSbS_2_ powders. The final product was collected for further analysis after the system was slowly cooled to room temperature. In addition, AgSbS_2_ thin films were also deposited by spin coating technique using the same complexes, as per the synthesis of AgSbS_2_ powders. Full details of thin film deposition and characterisation are presented in Sect. 3 of ESI.

### Materials characterisation

A Specac single reflectance ATR instrument (4000–400 cm^−1^) with resolution 4 cm^−1^ was used to record the infrared spectra (IR). Melting points of the complexes were obtained using a Barloworld SMP10. ^13^C NMR spectra were obtained using a Bruker AC400 FT-NMR spectrometer. Elemental analysis was performed with a Carlo Erba EA 1108 instrument. Thermogravimetric analysis (TGA), was performed using a Seiko SSC/S200 at a heating rate of 10 °C min^−1^ under nitrogen. Powder X-ray diffraction (XRD) measurements were carried out by a Bruker Xpert diffractometer, utilising Cu-Ka radiation (1.5406 Å). Raman spectra were recorded using a Renishaw 1000 microscope system equipped with laser excitation of 514 nm. Scanning electron microscopy (SEM) images were obtained using a Tescan SC Oxford SEM. Electrical properties of the thin films were measured using the Van Der Pauw method by means of a custom-build Hall effect measurement system.

## Supplementary information

Supplementary Information.
