# Supplementary material for: Direct synthesis of nanostructured silver antimony sulfide powders from metal xanthate precursors
Source: Sci Rep. 2021 Feb 4;11:3053. doi: 10.1038/s41598-021-82446-3 (PMC7862388; doi:10.1038/s41598-021-82446-3)
Supplement: Supplementary file 1 — Supplementary Information. [file 41598_2021_82446_MOESM1_ESM.docx]

**Supporting Information**

**Direct Synthesis of Nanostructured Silver Antimony Sulfide Powders from Metal Xanthate Precursors**

Yasser T. Alharbi,^1^ Firoz Alam,^1,2^ Abdelmajid Salhi^3^ Mohamed Missous^3^ and David J. Lewis^2^*

*^1^ Department of Chemistry, The University of Manchester, Oxford Road, Manchester M13 9PL, UK.*

*^2^ Department of Materials, The University of Manchester, Oxford Road, Manchester M13 9PL, UK.*

*^3^ Department of Electrical and Electronic Engineering, The University of Manchester, Sackville Street, Manchester, M13 9PL, UK*

*Correspondence and requests for materials should be addressed to D.J.L (email: david.lewis-4@manchester.ac.uk)

**Section 1: Precursors**


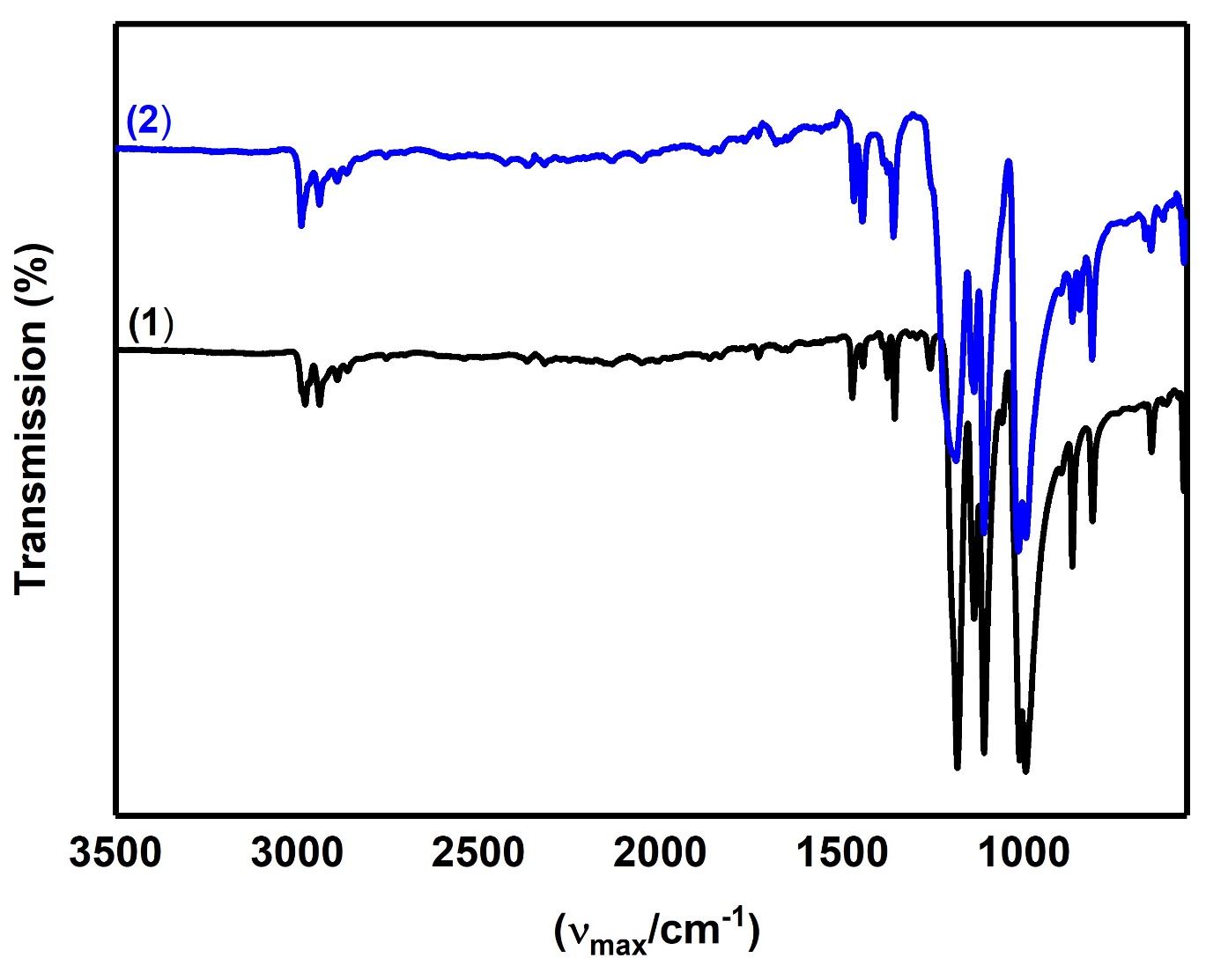


**Fig. S1.1** IR spectra of metal xanthate precursors [AgS_2_COEt] **(1)** and [Sb(S_2_COEt)_3_] **(2)**.

**Fig. S1.2** ^13^C NMR spectra of metal xanthates (a) [AgS_2_COEt] **(1)** and (b) [Sb(S_2_COEt)_3_] **(2)**.

**Section 2: Powders**


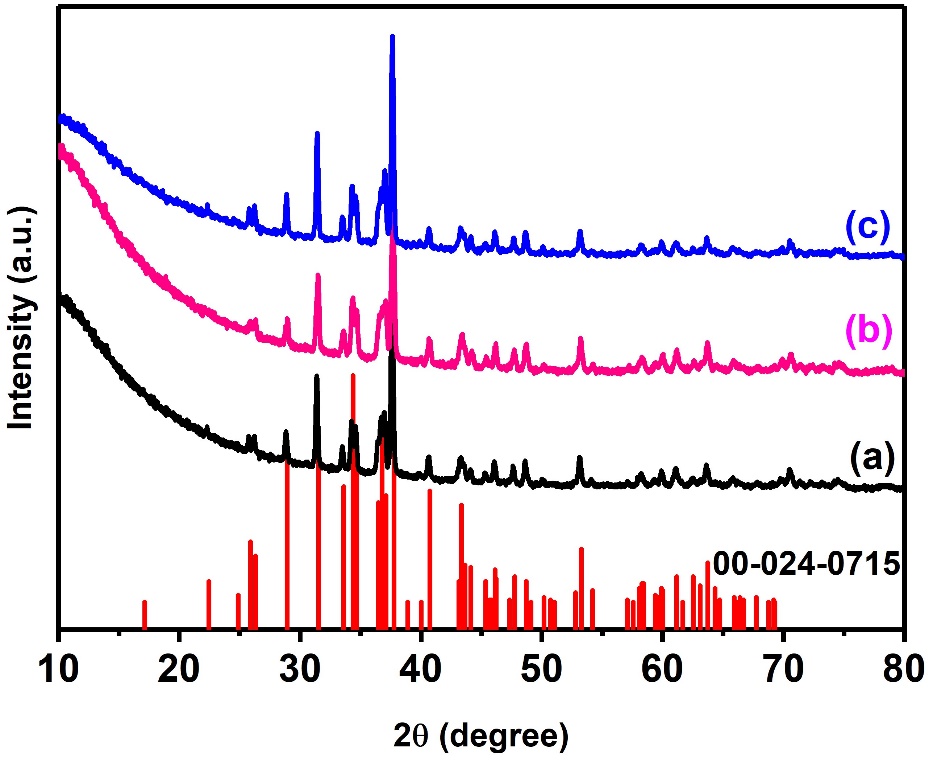


**Fig. S2.1** XRD powder patterns of pure phase Acanthite Ag_2_S produced from [AgS_2_COEt] (**1**) at different temperatures (a) 400 °C (b) 450 °C and (c) 500 °C for 1 h under nitrogen.


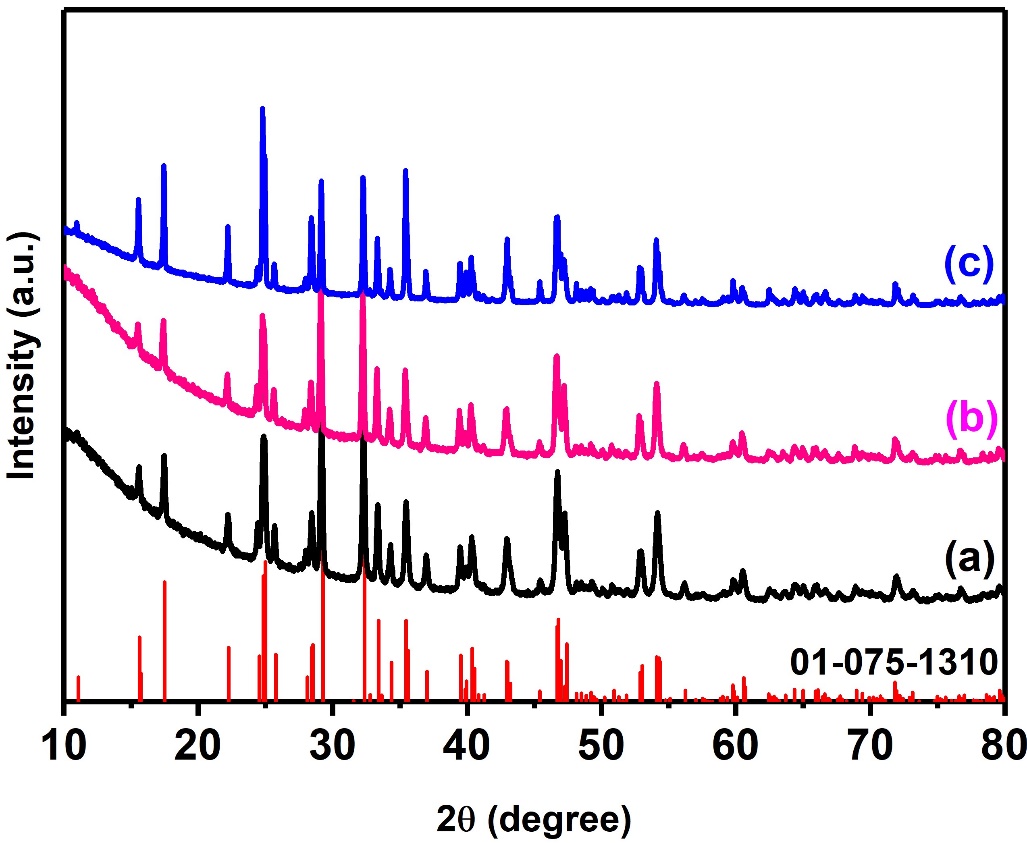


**Fig. S2.2** XRD powder patterns of Sb_2_S_3_ Stibnite phase produced from [Sb(S_2_COEt)_3_] at different temperatures (a) 400 °C (b) 450 °C and (c) 500 °C for 1 h under nitrogen.


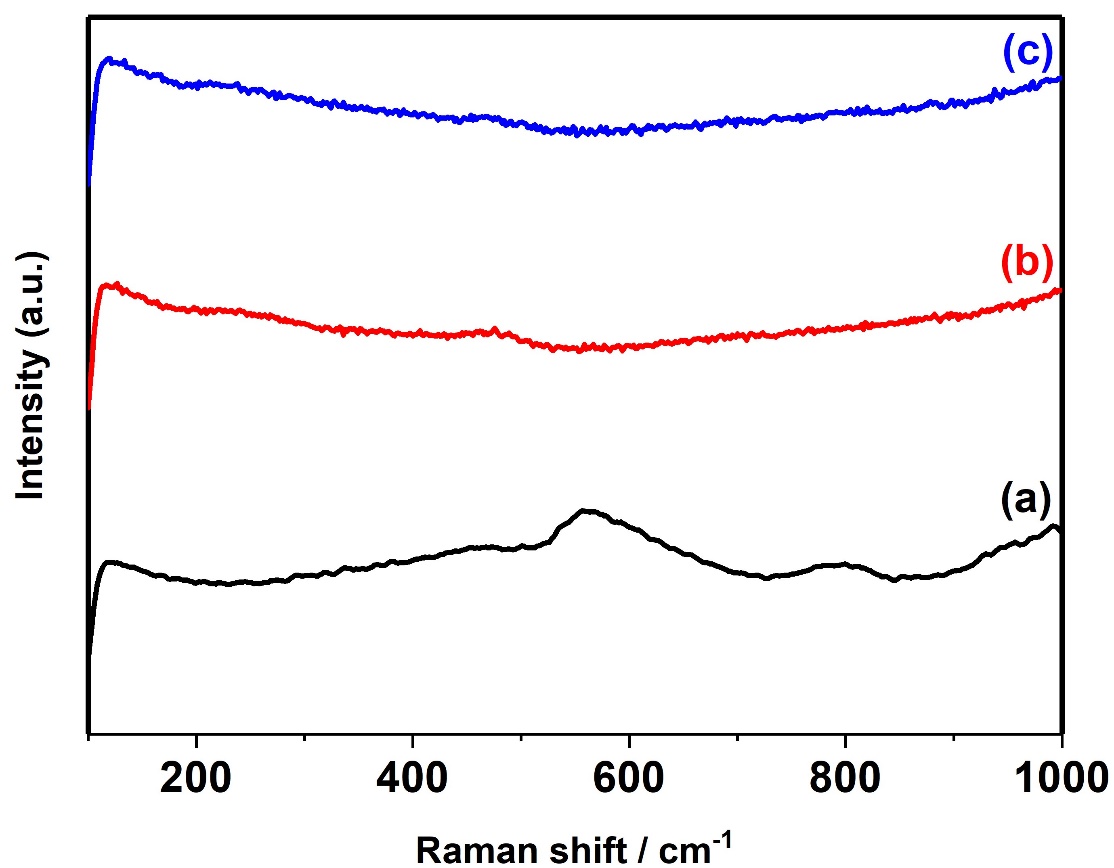


**Fig. S2.3** Raman spectra of Ag_2_S powders synthesised at various temperatures (a) 400 °C (b) 450 °C and (c) 500 °C for 1 h under nitrogen.


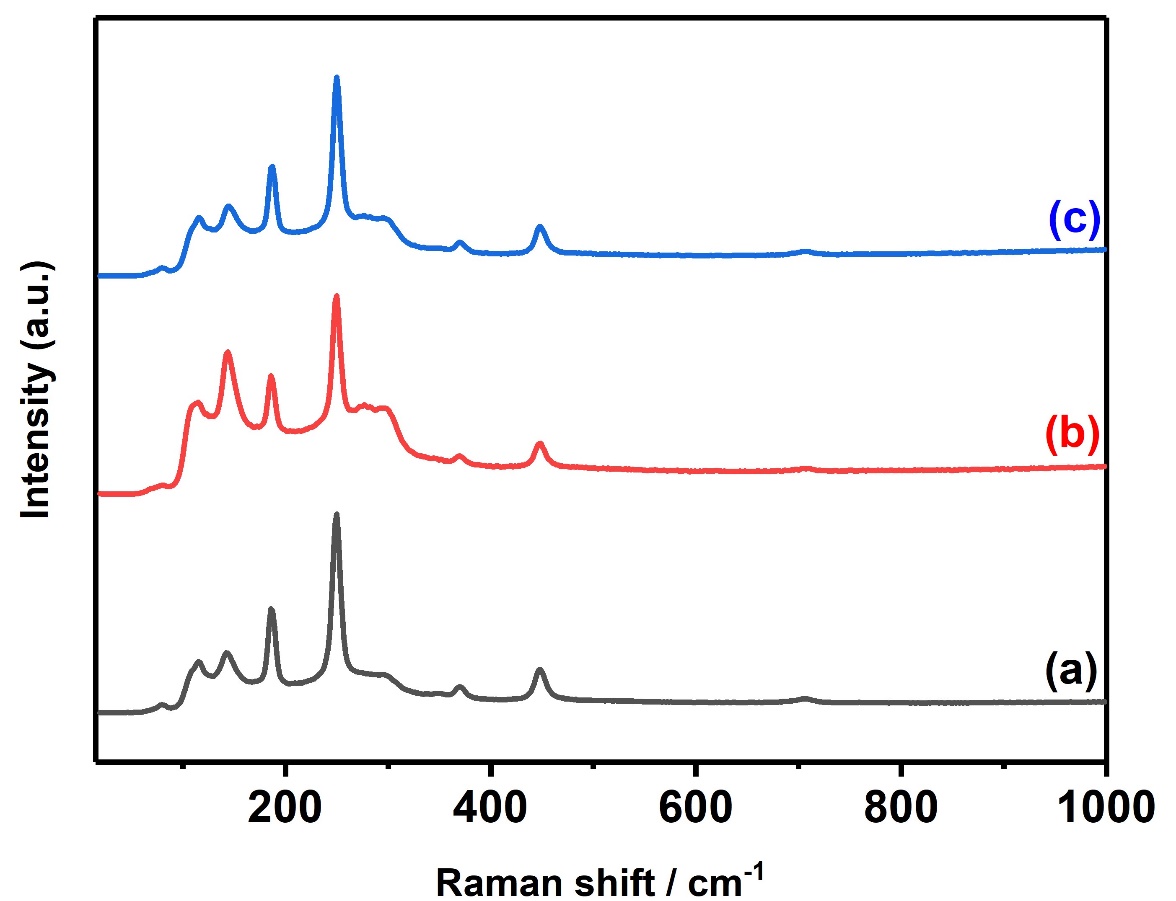


**Fig. S2.4** Raman spectra of Sb_2_S_3_ synthesised at different temperatures (a) 400 °C (b) 450 °C and (c) 500 °C for 1 h under nitrogen.

**
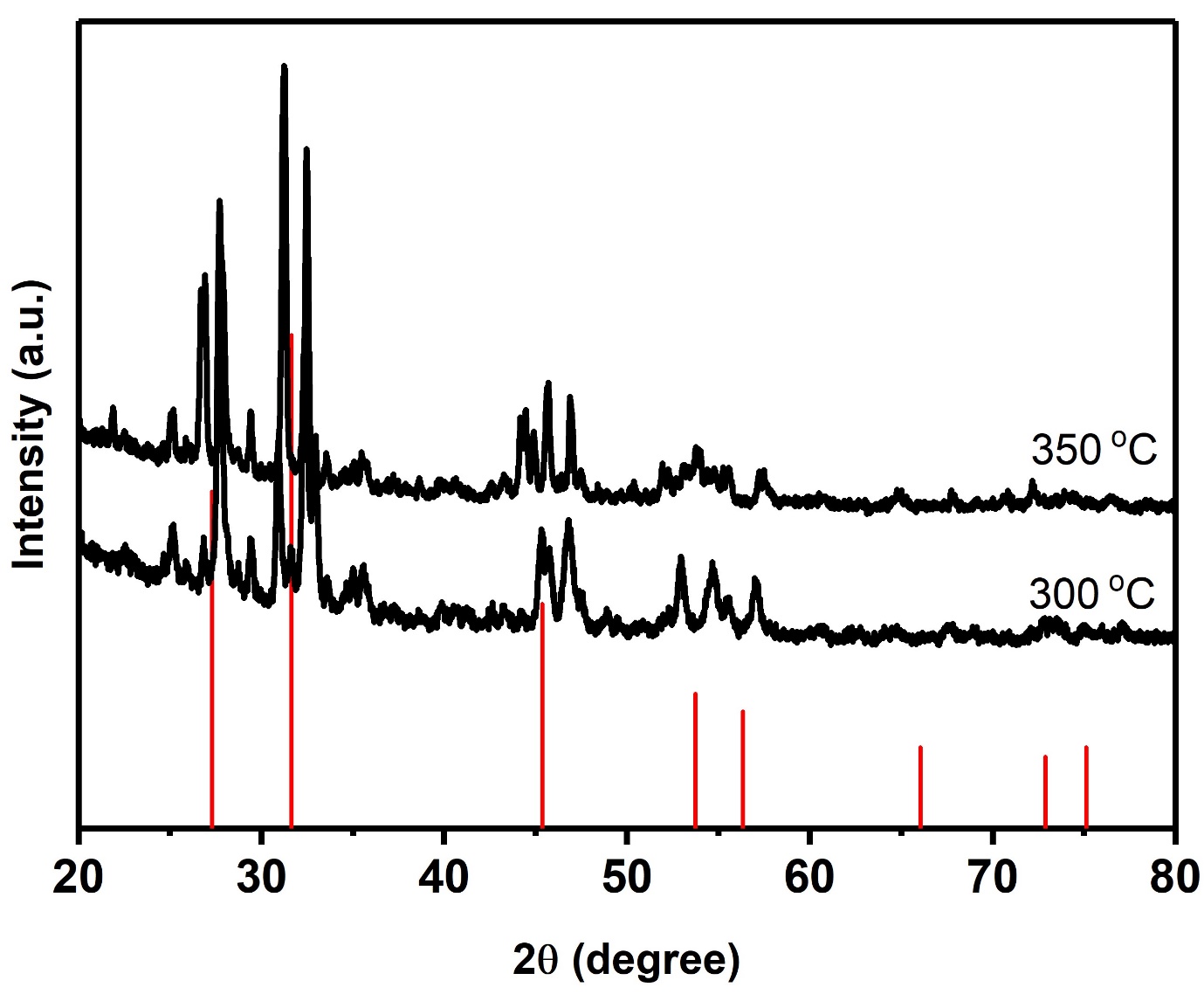
**

**Fig. S2.5** XRD pattern of AgSbS_2_ powders synthesised at 300 and 350 °C for 1h under nitrogen. The red sticks correspond to the standard powder diffraction pattern of cubic AgSbS_2_ (cuboargyrite, ICDD no. 00-017-0456).

.
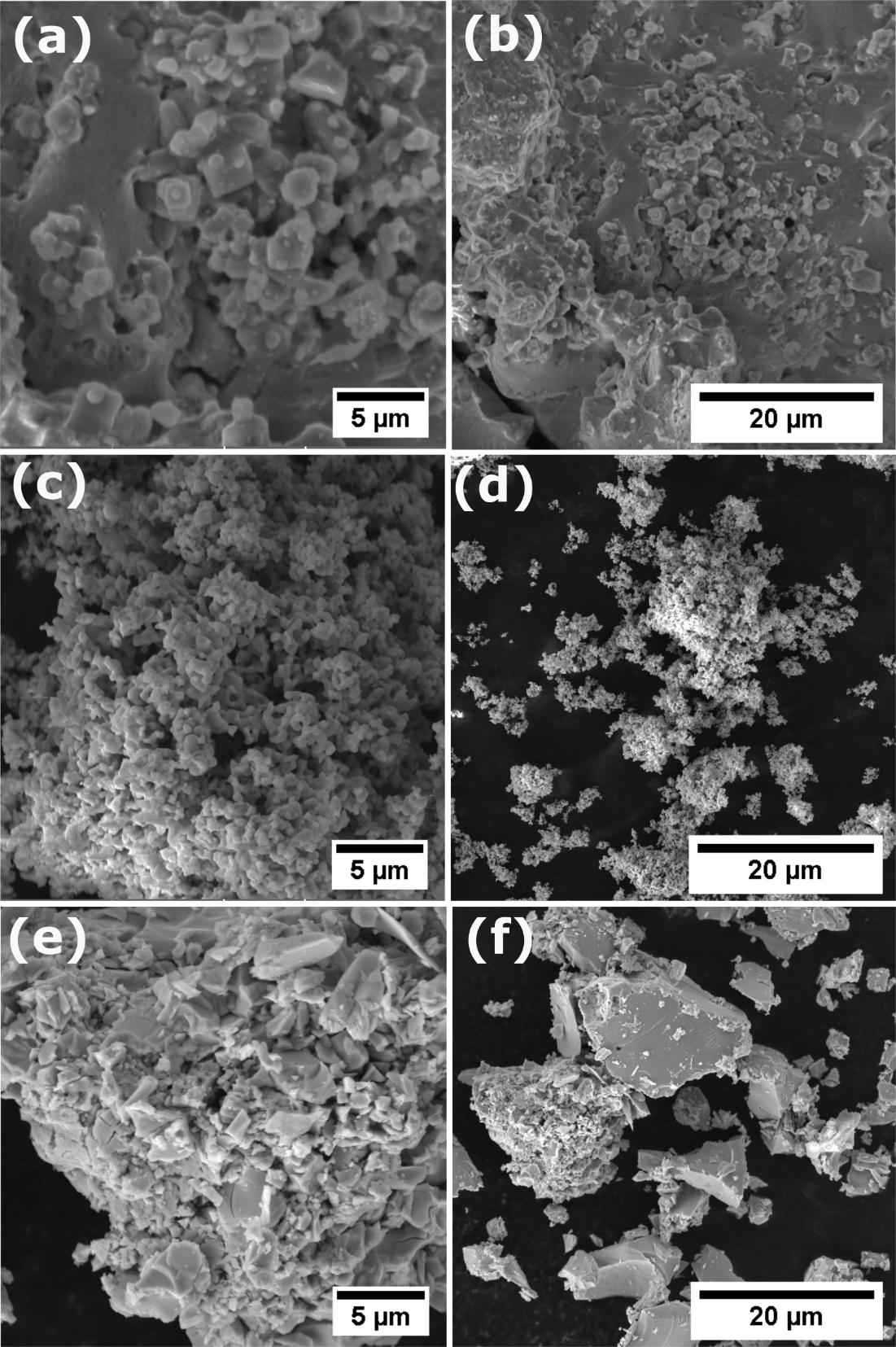


**Fig. S2.6** SEM images of AgSbS_2_ powders produced at different temperatures (a, b) 400°C (c, d) at 450°C and (e, f) at 500°C, respectively.


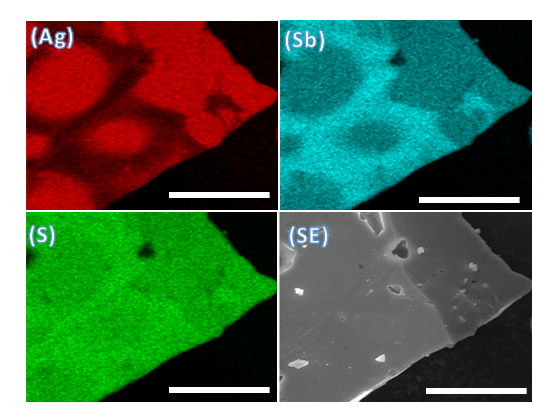


**Fig.S2.**7 EDX elemental mapping of AgSbS_2_ powder synthesised at 400 °C for 1 h showing the spatial distribution of Ag, Sb and S. Scale bars represent 10 µm in all cases. A secondary electron SEM image of the mapped area is included in each case, labelled as SE.


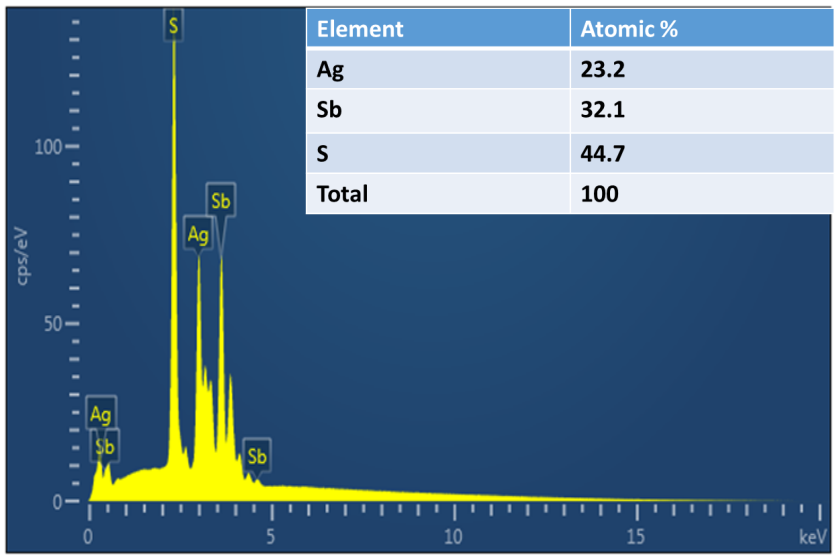


**Fig. S2.8** EDX spectrum showing the different atomic percentages of element present in AgSbS_2_ powder synthesised at 400 °C for 1 h under nitrogen.


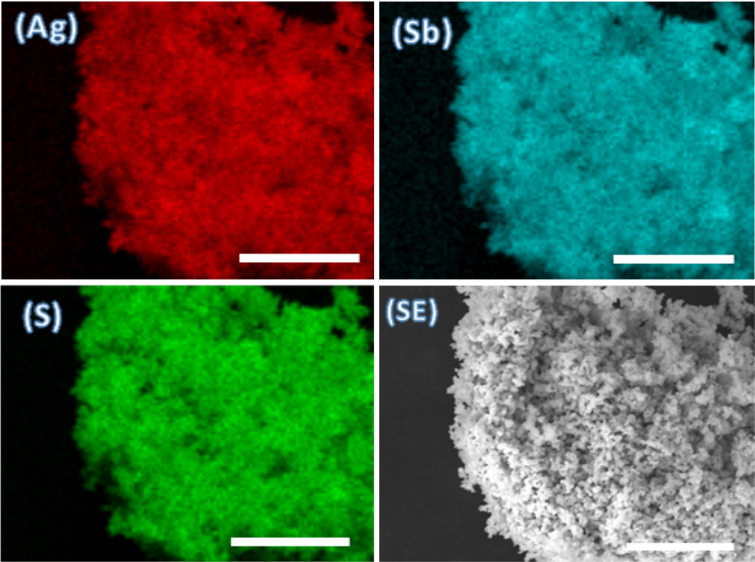


**Fig. S2.9** EDX elemental mapping of AgSbS_2_ powder synthesised at 450 °C for 1 h showing the spatial distribution of Ag, Sb and S. Scale bars represent 10 µm in all cases. A secondary electron SEM image of the mapped area is included in each case, labelled as SE.


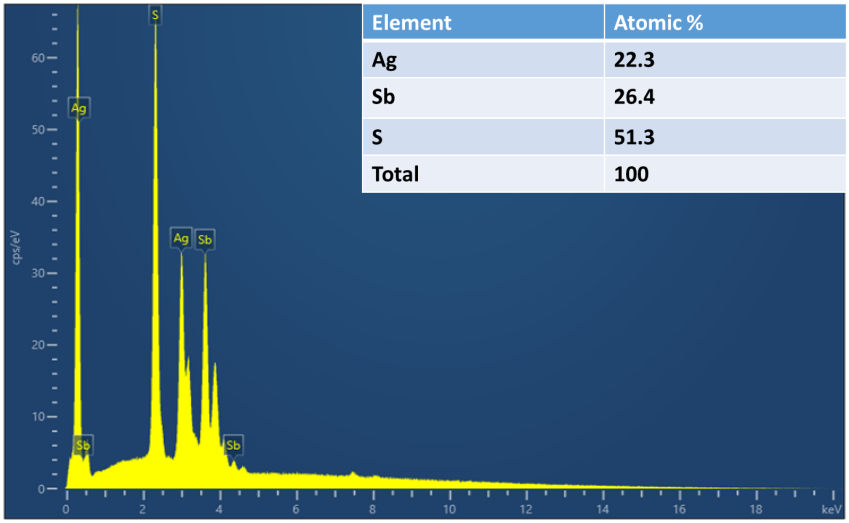


**Fig. S2.10** EDX spectrum showing the different atomic percentages of element present in AgSbS_2_ powder synthesised at 450 °C for 1h under nitrogen.

**
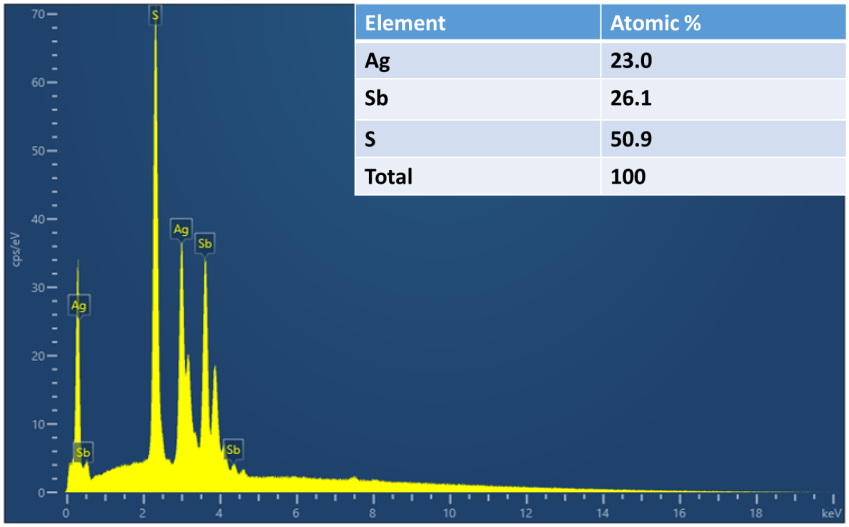
**

**Fig. S2.11** EDX spectrum showing the different atomic percentages of element present in AgSbS_2_ powder synthesised at 500 °C for 1h under nitrogen.

**Section 3: Thin films**

**Deposition of AgSbS_2_ thin films using spin coating technique:**

AgSbS_2_ thin film was deposited on the cleaned glass substrates of size 7×7mm^2^ by spin coating technique (Ossila, 24 V DC, 2.01 A) . The glass substrates were cleaned in soap solution, deionised water, acetone and isopropanol, 20 min. each using ultrasonicator. The mixture of 0.9 mmol of silver (I) ethylxanthate [AgS_2_COEt] **(1)** and 0.9 mmol antimony (III) ethylxanthate [Sb(S_2_COEt)_3_] **(2)** were dissolved in tetrahydrofuran (THF, 5 ml), which resulting in dark yellow solution. The as-obtained solution was dropped on to the glass substrate at room temperature using pipette and rotated at 1000 rpm for 20 s and allowed to dry at room temperature. The films were then placed into a tube furnace and heated at a temperature of 400 °C, 450 °C and 500 °C for 1 h under nitrogen atmosphere to produce the AgSbS_2_ thin film.

The crystal structures of AgSbS_2_ thin films were identified using powder X-ray diffraction (XRD). All the diffraction peaks with reflections of (1 1 1), (2 0 0), (2 2 0), (3 1 1), (2 2 2), (4 0 0), (3 3 1) and (4 2 0) corresponds to cubic phase of AgSbS_2_ (ICDD. No. 00-017-0456) with lattice parameters of a = 5.6520 Å, which is same as obtained for AgSbS_2_ powders. It is clear from the Fig. S3.1 that the peak intensity increases with increasing the annealing temperature from 400 to 500 °C.

The micro morphology of AgSbS_2_ thin films were studied by scanning electron microscopy (SEM). Micrographs of AgSbS_2_ thin films deposited by spin coating method from complexes (**1**) and (**2**) at growth temperatures of 400, 450 and 500 °C are shown in Fig. S3.2. SEM images show network of mesoporous architecture at all three temperatures. It can be clearly seen from Fig. S3.2 (e, f) that the particles are very well connected and make a network of mesoporous architecture.

The elemental maps revealed the uniform distribution of elements in films at various temperatures which illustrated in ESI (Fig. S3.3, S3.5 and S3.7. Elemental analysis using EDX spectroscopy of the AgSbS_2_ thin films agreed well with the expected 1:1:2 Ag:Sb:S stoichiometry of cuboargyrite as shown in Fig. S3.4, S3.6 and S3.8.


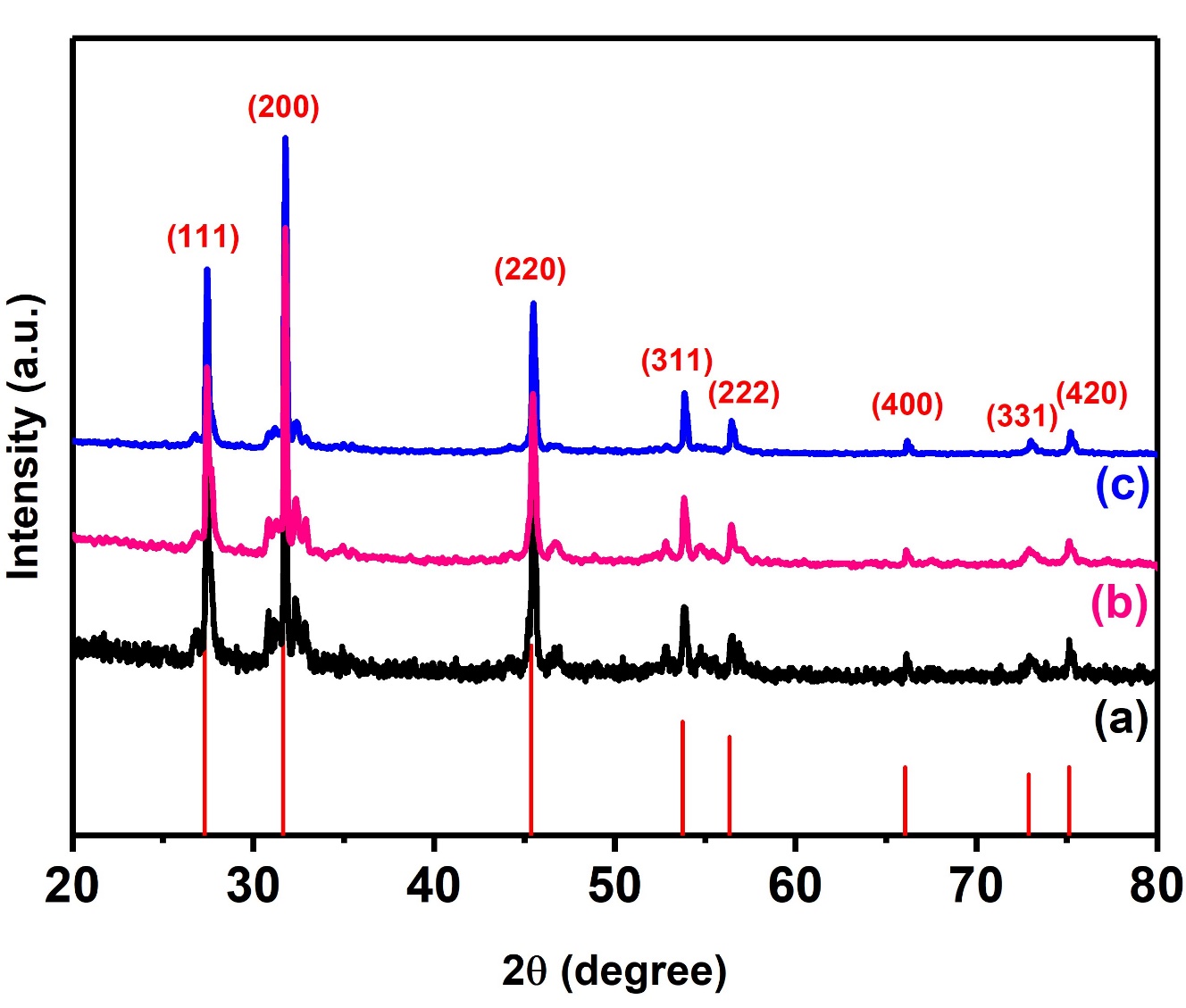


**Fig. S3.1** The XRD patterns of AgSbS_2_ thin films deposited using(**1**) and (**2**) and annealed at different temperature (a) 400 °C (b) 450°C and (c) 500°C, respectively for 1 h. The red stick correspond to the standard pattern of cuboargyrite (ICDD no. 00-017-0456).


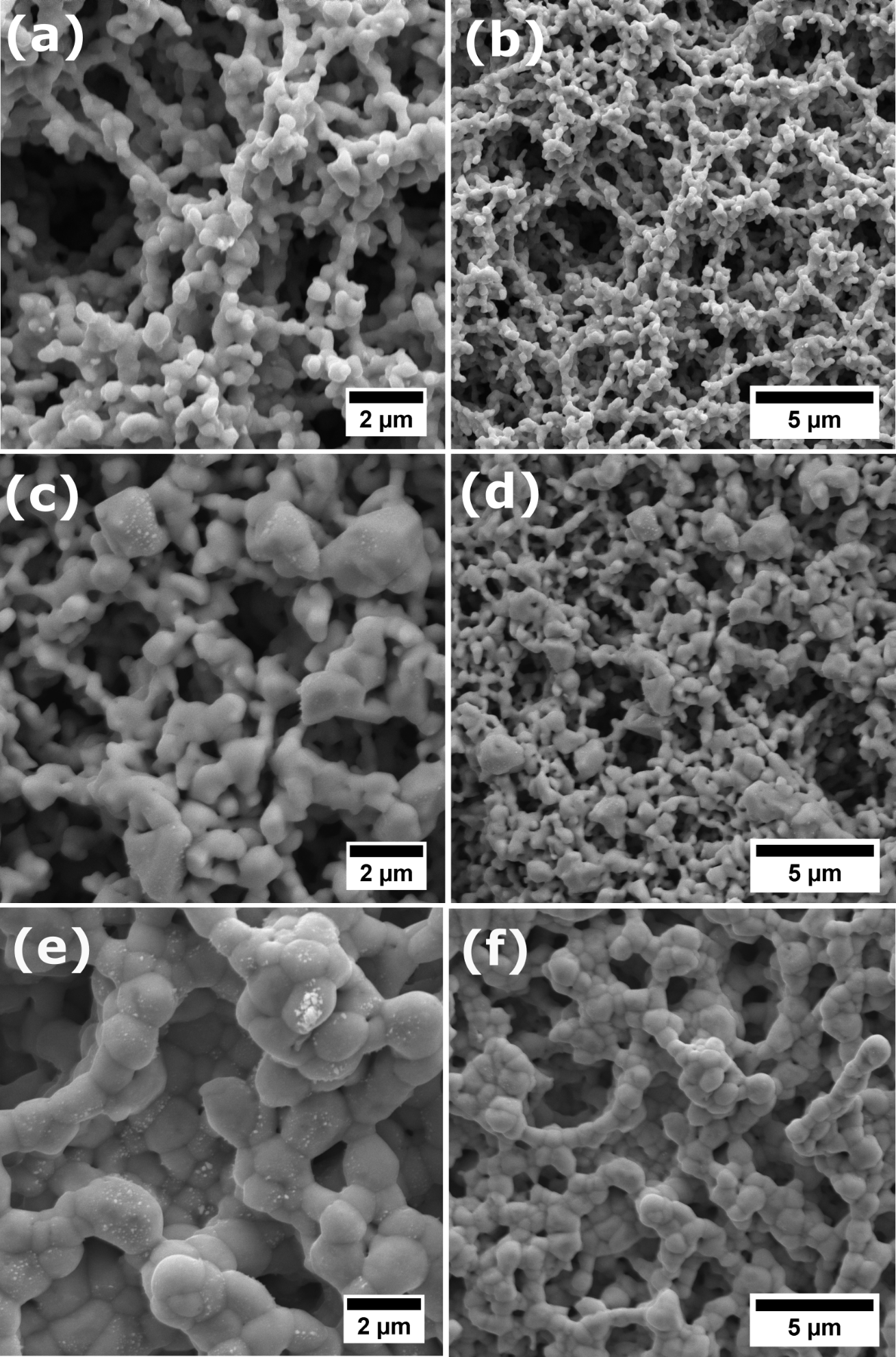


**Fig. S3.2** SEM images of AgSbS_2_ thin films deposited using (**1**) and (**2**) and annealed at different temperatures (a, b) 400 °C (c, d) at 450°C and (e, f) at 500°C, respectively.


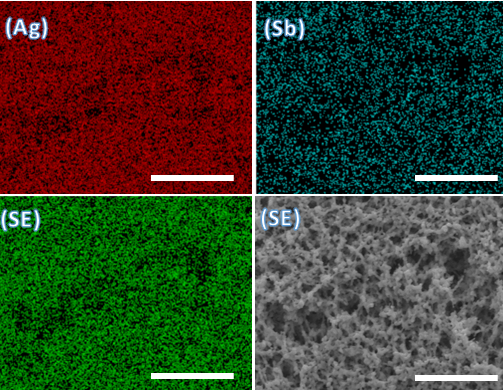


**Fig. S3.3** Elemental mapping of AgSbS_2_ thin film deposited from (**1**) and (**2**) and annealed at 400 °C for 1 h nitrogen, showing the distribution of Ag, Sb and S. Scale bars represent 5µm in all cases. A secondary electron SEM image of the mapped area is included in each case, labelled as SE.


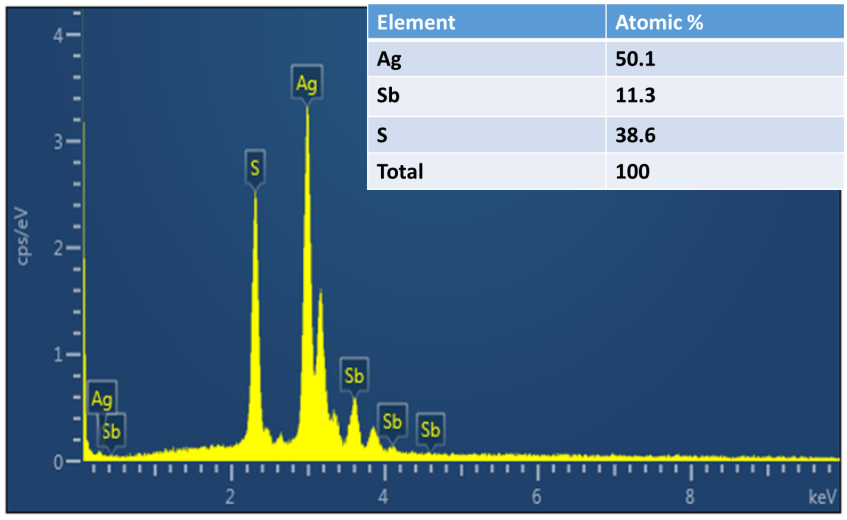


**Fig. S3.4** EDX spectrum of AgSbS_2_ thin film deposited from (**1**) and (**2**) and annealed at 400 °C for 1 h under nitrogen.


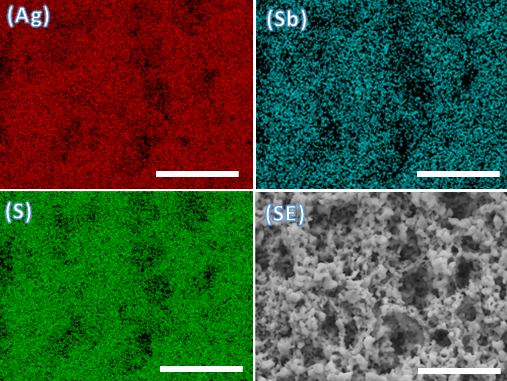


**Fig. S3.5** Elemental mapping of AgSbS_2_ thin film deposited from (**1**) and (**2**) and annealed at 450 °C for 1 h nitrogen, showing the distribution of Ag, Sb and S. Scale bars represent 5µm in all cases. A secondary electron SEM image of the mapped area is included in each case, labelled as SE.


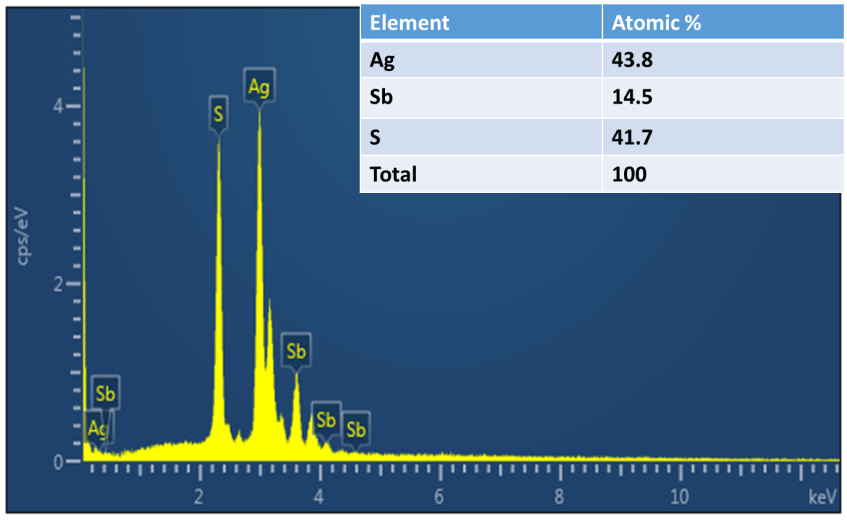


**Fig. S3.6** EDX spectrum of AgSbS_2_ thin film deposited from (**1**) and (**2**) and annealed at 450 °C for 1 h under nitrogen.


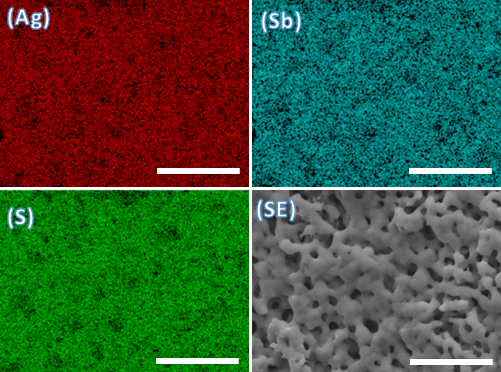


**Fig. S3.7** Elemental mapping of AgSbS_2_ thin film deposited from (**1**) and (**2**) and annealed at 500 °C for 1 h nitrogen, showing the distribution of Ag, Sb and S. Scale bars represent 5µm in all cases. A secondary electron SEM image of the mapped area is included in each case, labelled as SE.


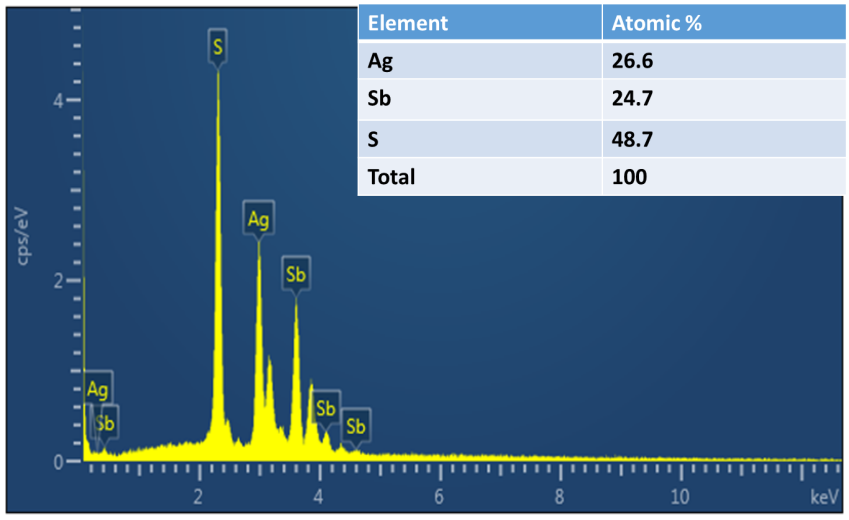


**Fig. S3.8** EDX spectrum of AgSbS_2_ thin film deposited from (**1**) and (**2**) and annealed at 450 °C for 1 h under nitrogen.
